# FGF21/adiponectin ratio predicts deterioration in glycemia: a 4.6-year prospective study in China

**DOI:** 10.1186/s12933-021-01351-1

**Published:** 2021-07-28

**Authors:** Dan Liu, Liang Wu, Qiongmei Gao, Xiaoxue Long, Xuhong Hou, Lingling Qian, Jiacheng Ni, Qichen Fang, Huating Li, Weiping Jia

**Affiliations:** 1 Department of Endocrinology and Metabolism, Shanghai Diabetes Institute, Shanghai Clinical Center for Diabetes, Shanghai Key Laboratory of Diabetes Mellitus, Shanghai Jiao Tong University Affiliated Sixth People’s Hospital, 600 Yishan Road, Shanghai, 200233 China; 2 Department of Medicine, Shanghai Jiao Tong University School of Medicine, Shanghai, China

**Keywords:** Fibroblast growth factor 21, Adiponectin, FGF21/Adiponectin ratio, Biomarker, New-onset diabetes, Prediabetes, Deterioration in glycemia

## Abstract

**Background:**

The fibroblast growth factor (FGF) 21-adiponectin pathway is involved in the regulation of insulin resistance. However, the relationship between the FGF21-adiponectin pathway and type 2 diabetes in humans is unclear. Here, we investigated the association of FGF21/adiponectin ratio with deterioration in glycemia in a prospective cohort study.

**Methods:**

We studied 6361 subjects recruited from the prospective Shanghai Nicheng Cohort Study in China. The association between baseline FGF21/adiponectin ratio and new-onset diabetes and incident prediabetes was evaluated using multiple logistic regression analysis.

**Results:**

At baseline, FGF21/adiponectin ratio levels increased progressively with the deterioration in glycemic control from normal glucose tolerance to prediabetes and diabetes (*p* for trend < 0.001). Over a median follow-up of 4.6 years, 195 subjects developed new-onset diabetes and 351 subjects developed incident prediabetes. Elevated baseline FGF21/adiponectin ratio was a significant predictor of new-onset diabetes independent of traditional risk factors, especially in subjects with prediabetes (odds ratio, 1.367; *p* = 0.001). Moreover, FGF21/adiponectin ratio predicted incident prediabetes (odds ratio, 1.185; *p* = 0.021) while neither FGF21 nor adiponectin were independent predictors of incident prediabetes (both *p* > 0.05). Furthermore, net reclassification improvement and integrated discrimination improvement analyses showed that FGF21/adiponectin ratio provided a better performance in diabetes risk prediction than the use of FGF21 or adiponectin alone.

**Conclusions:**

FGF21/adiponectin ratio independently predicted the onset of prediabetes and diabetes, with the potential to be a useful biomarker of deterioration in glycemia.

**Supplementary Information:**

The online version contains supplementary material available at 10.1186/s12933-021-01351-1.

## Introduction

Based on the obesity-promoting environment and genetic predisposition [1], type 2 diabetes mellitus has become an emerging health challenge worldwide and is estimated to affect 700 million people by 2045 [2]. Moreover, the global prevalence of impaired glucose tolerance, a high-risk state for diabetes development [3], is projected to reach 8.6% (548 million) by 2045 [2]. Circulating fibroblast growth factor (FGF) 21 and adiponectin have been demonstrated to be associated with obesity, insulin resistance (IR), and type 2 diabetes mellitus in cross-sectional [4–7] and prospective studies [8–12]. In view of the strikingly overlapping functions between FGF21 and adiponectin, the underlying role of the FGF21-adiponectin pathway in metabolic disorders has become a research hotspot.

Evidence from animal studies suggests that FGF21 stimulates the expression and secretion of adiponectin partially through the peroxisome proliferator-activated receptor γ in adipocytes, and that adiponectin is an essential mediator for the metabolic actions of FGF21 [13, 14]. However, circulating FGF21 levels are increased, whereas adiponectin concentrations are reduced in subjects with type 2 diabetes [8, 9, 15], suggesting dysfunction of the FGF21-adiponectin pathway. Considering the prognostic roles of FGF21 and adiponectin in diabetes, the relationship between the FGF21-adiponectin pathway dysfunction and diabetes development is unclear. Here, we investigated the association between FGF21/adiponectin ratio and deterioration in glycemia in a 4.6-year prospective cohort of 6361 Chinese subjects.

## Methods

### Study subjects

The Shanghai Nicheng Cohort study was a population-based, prospective study designed to assess the prevalence, incidence, and related factors of cardiometabolic diseases in China [16, 17]. The baseline survey was conducted between April 2013 and August 2014. The target population covered 23,375 residents aged 45–70 years who had lived in Nicheng County for at least 5 years, and 21,408 residents were invited verbally after excluding those who rejected (n = 1253) and could not be contacted (n = 714) [16]. A total of 17,212 participants completed the baseline survey, with an 80.4% response rate. From May to September 2018, subjects aged 55–70 years at baseline were invited to participate in the follow-up study (n = 10,075), and 7230 participants responded to the survey (aged 62.02 ± 3.91 years; proportion of males 43.2%). We excluded participants who received medications that may confound glycemia levels, including thiazide diuretics (n = 12), statins (n = 42), antipsychotics (n = 6), and glucocorticoids (n = 10), and those who were on insulin treatment that may influence adiponectin secretion (n = 112). Subjects without baseline serum samples for adiponectin and FGF21 measurements (n = 687) were also excluded. Thus, our subsequent measurements and analysis were conducted in 6361 subjects (aged 61.79 ± 3.99 years; proportion of males 42.6%) (Additional file 1: Figure S1). The study was approved by the ethics committee of the Shanghai Sixth People’s Hospital, following the principles of the Declaration of Helsinki. Written informed consent was obtained from all participants.

### Anthropometric and biochemical measurements

Details of biochemical measurements and anthropometric data collection, including body weight, height, body mass index (BMI), waist circumference, systolic blood pressure (SBP), and diastolic blood pressure (DBP), have been described previously [16, 17]. In brief, participants were asked to avoid high-intensity exercise and keep good rest on the day before the investigation. The baseline investigation took place in the morning after an overnight fasting. Upon arrival at the investigation site, subjects were arranged to rest for half an hour before blood collection. Information on demographics, history of diseases, family and medical history, smoking habits, and alcohol consumption was collected through a standard questionnaire. Blood samples collected in the fasting status were tested to measure the levels of fasting plasma glucose (FPG), fasting insulin (FINS), glycated hemoglobin A_1c_ (HbA_1c_), high-sensitivity C-reactive protein (hsCRP), total cholesterol, triglyceride, high-density lipoprotein cholesterol (HDL-C), and low-density lipoprotein cholesterol (LDL-C). Plasma glucose was determined using the glucose oxidase method. HbA_1c_ values were measured using high-performance liquid chromatography (VARIANT II, Bio-Rad Laboratories, Inc., Hercules, CA). Participants without a self-reported history of diabetes underwent a 75 g oral glucose tolerance test (OGTT) at baseline, while they were not administered in the 4.6-year follow-up survey. Basal insulin secretion and insulin sensitivity were estimated using the homeostasis model assessment (HOMA): HOMA-β (%) = [FINS (mU/L) × 6 × 3.33]/[FPG (mmol/L) − 3.5] and HOMA-IR = FINS (mU/L) × FPG (mmol/L)/22.5 [18, 19].

### Serum adiponectin and FGF21 measurements

We measured adiponectin and FGF21 levels in all 6361 subjects with baseline serum samples. Serum adiponectin levels were quantified using a latex particle-enhanced immunoturbidimetric assay (Antibody and Immunoassay Services, University of Hong Kong). In brief, a standard or serum sample was added to a cuvette and mixed with the reaction buffer containing salt and polyether compounds. After a short incubation, the suspension of microparticles coated with rabbit polyclonal antibody against human adiponectin was added to the cuvette and mixed. The extent to which the microparticle aggregates were quantified by the amount of light scattering measured as absorbance using a chemistry analyzer. The concentration of adiponectin in unknown samples was interpolated from a reference curve using the standards provided. The lowest concentration of adiponectin that could be detected by this assay was 1.0 μg/mL. The intra- and inter-assay variations in adiponectin measurement were 2.77% and 8.42%, respectively. Baseline FGF21 concentrations were quantified using ELISA kits (Antibody and Immunoassay Services, University of Hong Kong) [19]. The lowest concentration of FGF21 detected in this assay was 30 pg/mL. The intra- and inter-assay variations in FGF21 measurement were 6.16% and 8.55%, respectively.

### Definitions

According to the World Health Organization 1999 criteria [20] and the standards of medical care in diabetes proposed by the American Diabetes Association in 2018 [21], categories of hyperglycemia were defined as follows: prediabetes (6.1 mmol/L ≤ FPG < 7.0 mmol/L, 7.8 mmol/L ≤ 2-h plasma glucose [2hPG] < 11.1 mmol/L, and/or 5.7% ≤ HbA_1c_ < 6.5%), and diabetes mellitus (a self-reported history of diabetes, FPG ≥ 7.0 mmol/L, 2hPG ≥ 11.1 mmol/L, and/or HbA_1c_ ≥ 6.5%).

### Statistical analysis

All statistical analyses were performed using SPSS version 25.0 (IBM, Armonk, NY) and R software 3.6.2 (Package PredictABEL). Data are expressed as mean ± SD, median (interquartile range), and percentage. Data with skewed distribution, as determined by the Kolmogorov–Smirnov test, were natural logarithmically transformed (log_e_-transformed) before analysis. Variables were compared between groups by Student’s unpaired t test, or one-way analysis of variance for continuous data, and chi-squared tests for categorical data, respectively. The baseline and follow-up glucose metabolism of the participants were compared by the paired t-test. The optimal cut-off values of biomarkers were identified by calculating the Youden index (sensitivity + specificity − 1). Baseline variables that were significantly different between subjects with and without new-onset diabetes or were biologically relevant to diabetes were included in the logistic regression analysis. The association of FGF21/adiponectin ratio as a continuous variable with the risk of diabetes was examined using multiple logistic regression analysis. The odds ratio (OR) for FGF21/adiponectin ratio was estimated as per SD increase in log_e_-transformed. Additionally, the association of FGF21/adiponectin ratio, as a categorical variable (above the cutoff value), with new-onset diabetes was also analyzed. The performance of models in diabetes risk prediction was assessed using C statistics, net reclassification improvement (NRI), and integrated discrimination improvement (IDI). Moreover, we performed subgroup analyses to further investigate the association of FGF21/adiponectin ratio with incident prediabetes, new-onset diabetes, and deterioration in glycemia. In all statistical tests, two-sided *p* values < 0.05 were considered statistically significant.

## Results

### Baseline FGF21/adiponectin ratio levels increased with the deterioration in glycemia at baseline

A total of 6361 participants were included in our study. Among them, 1553 had normal glucose tolerance (NGT), 3244 had prediabetes, and 1564 subjects had diabetes at baseline (Table 1). Subjects with prediabetes and diabetes were older and had higher BMI, waist circumference, SBP, DBP, total cholesterol, triglyceride, hsCRP, FPG, 2hPG, HbA_1c_, FINS, and lower HDL-C levels (all *p* for trend < 0.001). Serum FGF21 levels and FGF21/adiponectin ratio increased gradually with the deterioration in glycemic control from NGT to prediabetes and diabetes, while serum adiponectin concentrations progressively decreased (all *p* for trend < 0.001).

**Table 1:** Baseline characteristics of subjects with normal glucose tolerance, prediabetes, and type 2 diabetes (n = 6361)

	Normal glucose tolerance	Prediabetes	Type 2 diabetes	*p* for trend
n	1553	3244	1564	
Males (%)	47.84	42.05	38.49	< 0.001
Age (years)	61.30 ± 3.91	61.83 ± 3.99	62.20 ± 4.04	< 0.001
Current smoker (%)	25.05	20.65	18.16	< 0.001
Current Drinker (%)	16.29	14.98	14.58	0.183
BMI (kg/m^2^)	23.97 ± 3.00	24.93 ± 3.23	25.98 ± 3.43	< 0.001
Waist circumference (cm)	82.81 ± 9.02	84.78 ± 9.29	87.60 ± 9.29	< 0.001
Systolic blood pressure (mmHg)	132.33 ± 15.60	135.56 ± 16.42	139.71 ± 17.19	< 0.001
Diastolic blood pressure (mmHg)	83.27 ± 8.88	84.46 ± 8.54	85.35 ± 8.50	< 0.001
Total cholesterol (mmol/L)	5.04 ± 0.95	5.28 ± 0.97	5.38 ± 1.06	< 0.001
Triglyceride (mmol/L)^a^	1.15 (0.81,1.62)	1.36 (0.96,1.97)	1.61 (1.14,2.40)	< 0.001
HDL-C (mmol/L)^a^	1.34 (1.10,1.60)	1.30 (1.09,1.52)	1.25 (1.06,1.49)	< 0.001
LDL-C (mmol/L)	2.97 ± 0.76	3.20 ± 0.80	3.25 ± 0.87	< 0.001
hsCRP (μg/mL)^a^	0.69 (0.37,1.39)	0.88 (0.46,1.75)	1.17 (0.59,2.46)	< 0.001
Fasting plasma glucose (mmol/L)	5.48 ± 0.38	5.89 ± 0.49	7.73 ± 1.95	< 0.001
2-h plasma glucose (mmol/L)	6.19 ± 1.08	7.83 ± 1.69	14.39 ± 4.49	< 0.001
Fasting insulin (μU/mL)^a^	5.48 (4.01,7.64)	6.95 (4.95,9.76)	8.28 (5.75,12.15)	< 0.001
HOMA-β (%)^a^	55.53 (40.88,76.38)	59.27 (40.98,84.30)	43.45 (27.50,66.92)	< 0.001
HOMA-IR^a^	1.35 (0.96,1.89)	1.82 (1.29,2.57)	2.72 (1.87,4.16)	< 0.001
HbA_1c_ (mmol/mol)	34.58 ± 2.85	38.82 ± 3.64	50.29 ± 19.72	< 0.001
HbA_1c_ (%)	5.31 ± 0.26	5.70 ± 0.33	6.75 ± 1.80	< 0.001
FGF21 (pg/mL)^a^	202.78 (120.33,308.17)	236.80 (144.88,362.09)	261.07 (167.62,398.87)	< 0.001
Adiponectin (μg/mL)^a^	4.51 (3.34,6.00)	4.06 (3.08,5.40)	3.54 (2.70,4.72)	< 0.001
FGF21/adiponectin ratio (pg/μg)^*a*^	45.37 (23.84,78.71)	58.52 (31.99,100.53)	73.92 (40.47,128.40)	< 0.001

Data are presented as mean ± SD or median (interquartile range)

BMI: body mass index; FGF21: fibroblast growth factor 21; HDL-C: high-density lipoprotein cholesterol; HOMA: homeostasis model assessment; hsCRP: high-sensitivity C-reactive protein; IR: insulin resistance; LDL-C: low-density lipoprotein cholesterol

^a^Log_e_-transformed before analysis

Serum FGF21/adiponectin ratio was positively correlated with BMI, waist circumference, triglyceride, hsCRP, FINS, and HOMA-IR, but negatively correlated with HDL-C (all *p* < 0.001) (Additional file 1: Table S1). Correlations between FGF21/adiponectin ratio and triglyceride and HDL-C remained significant after adjustment for age and BMI (Additional file 1: Table S1).

### Baseline FGF21/adiponectin ratio levels were significantly higher in subjects with new-onset diabetes

Over a median follow-up of 4.6 years (interquartile range: 4.3–4.8), 195 subjects developed new-onset diabetes and 351 subjects developed incident prediabetes (Additional file 1: Figure S1). The baseline and follow-up glucose metabolism of the participants are summarized in Additional file 1: Table S2. Table 2 shows the baseline characteristics of the subjects with and without new-onset diabetes. Participants who developed diabetes had higher BMI, waist circumference, blood pressure, triglyceride, hsCRP, FPG, 2hPG, HbA_1c_, FINS, HOMA-IR, and lower HDL-C levels than those who did not develop diabetes (all *p* < 0.001). They also had a higher proportion of prediabetes at baseline (*p* < 0.001). Circulating FGF21/adiponectin ratio (median 85.49 pg/μg vs. 52.78 pg/μg, *p* < 0.001) and FGF21 levels (median 304.08 pg/mL *vs*. 223.98 pg/mL, *p* < 0.001) were higher, but adiponectin concentrations (median 3.50 μg/mL vs. 4.21 μg/mL, *p* < 0.001) were lower in participants with new-onset diabetes. Subjects with incident prediabetes were older and had higher baseline FPG, 2hPG, and HbA_1c_ levels than those without (Additional file 1: Table S3). Using the Youden index, the optimal cutoff values of serum biomarkers for new-onset diabetes were as follows: 73.96 pg/μg in males and 80.63 pg/μg in females for FGF21/adiponectin ratio; 393.59 pg/mL for FGF21; 3.06 μg/mL in males and 4.20 μg/mL in females for adiponectin.

**Table 2:** Baseline characteristics of subjects with or without new-onset diabetes at 4.6 years (n = 4797)

	Without new-onset diabetes	With new-onset diabetes	*p* value
n	4602	195	
Males (%)	43.79	47.18	0.350
Age (years)	61.65 ± 3.97	62.01 ± 3.96	0.212
Current smoker (%)	21.84	27.69	0.054
Current Drinker (%)	15.49	13.33	0.413
BMI (kg/m^2^)	24.56 ± 3.17	26.02 ± 3.25	< 0.001
Waist circumference (cm)	83.98 ± 9.25	87.96 ± 8.52	< 0.001
Systolic blood pressure (mmHg)	134.26 ± 16.13	140.38 ± 17.38	< 0.001
Diastolic blood pressure (mmHg)	83.99 ± 8.66	86.22 ± 8.74	< 0.001
Total cholesterol (mmol/L)	5.20 ± 0.97	5.24 ± 1.02	0.582
Triglyceride (mmol/L)^a^	1.26 (0.89,1.84)	1.55 (1.04,2.11)	< 0.001
HDL-C (mmol/L)^*a*^	1.32 (1.09,1.56)	1.22 (0.99,1.40)	< 0.001
LDL-C (mmol/L)	3.13 ± 0.79	3.19 ± 0.84	0.237
hsCRP (μg/mL)^a^	0.80 (0.42,1.61)	1.19 (0.73,2.26)	< 0.001
Fasting plasma glucose (mmol/L)	5.74 ± 0.49	6.20 ± 0.46	< 0.001
2-h plasma glucose (mmol/L)	7.25 ± 1.69	8.36 ± 1.69	< 0.001
Prediabetes (%)	66.56	92.82	< 0.001
Fasting insulin (μU/mL)^a^	6.38 (4.58,8.97)	7.91 (5.51,11.27)	< 0.001
HOMA-β (%)^a^	57.81 (40.99,81.52)	60.28 (39.60,84.53)	0.802
HOMA-IR^a^	1.63 (1.14,2.33)	2.16 (1.48,3.22)	< 0.001
HbA_1c_ (mmol/mol)	37.28 ± 3.85	41.43 ± 3.97	< 0.001
HbA_1c_ (%)	5.56 ± 0.35	5.94 ± 0.36	< 0.001
FGF21 (pg/mL)^a^	223.98 (135.62,342.64)	304.08 (183.70,493.50)	< 0.001
Adiponectin (μg/mL)^a^	4.21 (3.20,5.63)	3.50 (2.60,4.65)	< 0.001
FGF21/adiponectin ratio (pg/μg)^a^	52.78 (28.77,91.88)	85.49 (41.72,166.18)	< 0.001

Data are presented as mean ± SD or median (interquartile range)

BMI: body mass index; FGF21: fibroblast growth factor 21; HDL-C: high-density lipoprotein cholesterol; HOMA: homeostasis model assessment; hsCRP: high-sensitivity C-reactive protein; IR: insulin resistance; LDL-C: low-density lipoprotein cholesterol

^a^Log_e_-transformed before analysis

### Baseline FGF21/adiponectin ratio levels independently predicted deterioration in glycemia

Independent predictors of new-onset diabetes were identified using multiple logistic regression analysis, adjusted for age, BMI, waist circumference, SBP, triglyceride, HDL-C, LDL-C, hsCRP, sex, HOMA-IR, FPG, and FGF21/adiponectin ratio. As shown in Table 3, baseline FGF21/adiponectin ratio was significantly associated with new-onset diabetes (OR 1.45 [95% confidence interval, CI 1.21–1.74], *p* < 0.001), together with SBP, triglyceride, HDL-C, hsCRP, and FPG levels (all *p* < 0.05) (Model 1A). FGF21/adiponectin ratio remained an independent predictor of new-onset diabetes when baseline 2hPG or HbA_1c_ levels were involved in the model (Model 2A and 3A). Furthermore, the positive association of FGF21/adiponectin ratio with new-onset diabetes remained significant (OR 1.93 [95% CI 1.38–2.69], *p* < 0.001) when FGF21/adiponectin ratio was treated as a categorical variable, and serum FGF21/adiponectin ratio levels independently predicted new-onset diabetes in both males and females (Additional file 1: Table S4).

**Table 3:** Baseline parameters predictive of new-onset diabetes over 4.6 years, examined using multiple logistic regression (n = 4797)

	Model 1			Model 2			Model 3		
	A	B	C	A	B	C	A	B	C
Age	1.04 (0.89–1.21)	1.02 (0.88–1.19)	1.03 (0.88–1.19)	1.05 (0.90–1.22)	1.03 (0.88–1.20)	1.03 (0.89–1.20)	1.03 (0.88–1.20)	1.01 (0.87–1.18)	1.01 (0.87–1.18)
BMI	1.25 (0.98–1.59)	1.25 (0.98–1.59)	1.25 (0.99–1.59)	1.05 (0.82–1.33)	1.05 (0.82–1.33)	1.05 (0.83–1.32)	1.01 (0.79–1.29)	1.00 (0.79–1.28)	1.02 (0.81–1.29)
Waist circumference	1.01 (0.78–1.30)	1.01 (0.79–1.31)	1.04 (0.81–1.33)	1.08 (0.84–1.39)	1.08 (0.84–1.39)	1.10 (0.87–1.40)	1.05 (0.82–1.35)	1.06 (0.82–1.36)	1.06 (0.83–1.35)
SBP	1.17 (1.00–1.36)	1.16 (1.00–1.36)	1.18 (1.02–1.37)	1.23 (1.06–1.43)	1.23 (1.06–1.42)	1.25 (1.08–1.44)	1.27 (1.09–1.47)	1.26 (1.08–1.47)	1.28 (1.10–1.48)
Triglyceride^a^	0.81 (0.66–0.99)	0.82 (0.67–1.00)	0.89 (0.74–1.08)	0.79 (0.64–0.96)	0.79 (0.64–0.97)	0.88 (0.72–1.06)	0.82 (0.66–1.01)	0.82 (0.67–1.01)	0.91 (0.75–1.10)
HDL-C^a^	0.66 (0.53–0.82)	0.63 (0.51–0.78)	0.67 (0.54–0.83)	0.79 (0.63–0.98)	0.76 (0.61–0.94)	0.79 (0.64–0.97)	0.78 (0.62–0.97)	0.75 (0.60–0.93)	0.79 (0.63–0.98)
LDL-C	1.14 (0.96–1.35)	1.14 (0.96–1.35)	1.09 (0.93–1.29)	1.05 (0.89–1.25)	1.06 (0.89–1.25)	1.02 (0.87–1.20)	0.95 (0.80–1.13)	0.96 (0.81–1.14)	0.92 (0.78–1.08)
hsCRP^a^	1.20 (1.02–1.41)	1.21 (1.03–1.42)	1.22 (1.05–1.43)	1.14 (0.97–1.33)	1.15 (0.98–1.34)	1.16 (1.00–1.35)	1.11 (0.94–1.32)	1.12 (0.95–1.32)	1.14 (0.97–1.34)
Sex	1.06 (0.75–1.50)	0.97 (0.68–1.37)	1.30 (0.91–1.85)	0.71 (0.51–1.00)	0.65 (0.46–0.91)	0.81 (0.58–1.15)	0.67 (0.47–0.95)	0.61 (0.43–0.87)	0.77 (0.54–1.09)
HOMA-IR^a^	0.89 (0.72–1.10)	0.92 (0.74–1.14)	0.85 (0.69–1.04)	1.23 (1.01–1.49)	1.27 (1.05–1.54)	1.22 (1.00–1.48)	1.20 (0.98–1.47)	1.24 (1.01–1.52)	1.18 (0.97–1.45)
FPG	2.67 (2.23–3.20)	2.67 (2.23–3.19)	2.76 (2.32–3.29)						
2hPG				1.79 (1.52–2.12)	1.81 (1.53–2.14)	1.75 (1.49–2.05)			
HbA_1c_							3.45 (2.82–4.21)	3.46 (2.84–4.23)	3.47 (2.87–4.21)
FGF21/adiponectin ratio^a^	1.45 (1.21–1.74)			1.42 (1.19–1.69)			1.36 (1.13–1.63)		
FGF21^a^		1.36 (1.15–1.61)			1.37 (1.15–1.62)			1.31 (1.10–1.56)	
Adiponectin^a^			0.80 (0.67–0.96)			0.87 (0.73–1.03)			0.87 (0.73–1.04)

Data are presented as OR (95% CI). ORs were estimated as per SD increase

2hPG: 2-h plasma glucose; BMI: body mass index; FGF21: fibroblast growth factor 21; FPG: fasting plasma glucose; HDL-C: high-density lipoprotein cholesterol; HOMA-IR: homeostasis model assessment of insulin resistance; hsCRP: high-sensitivity C-reactive protein; LDL-C: low-density lipoprotein cholesterol; SBP: systolic blood pressure

^a^Log_e_-transformed before analysis

**Fig. 1:**
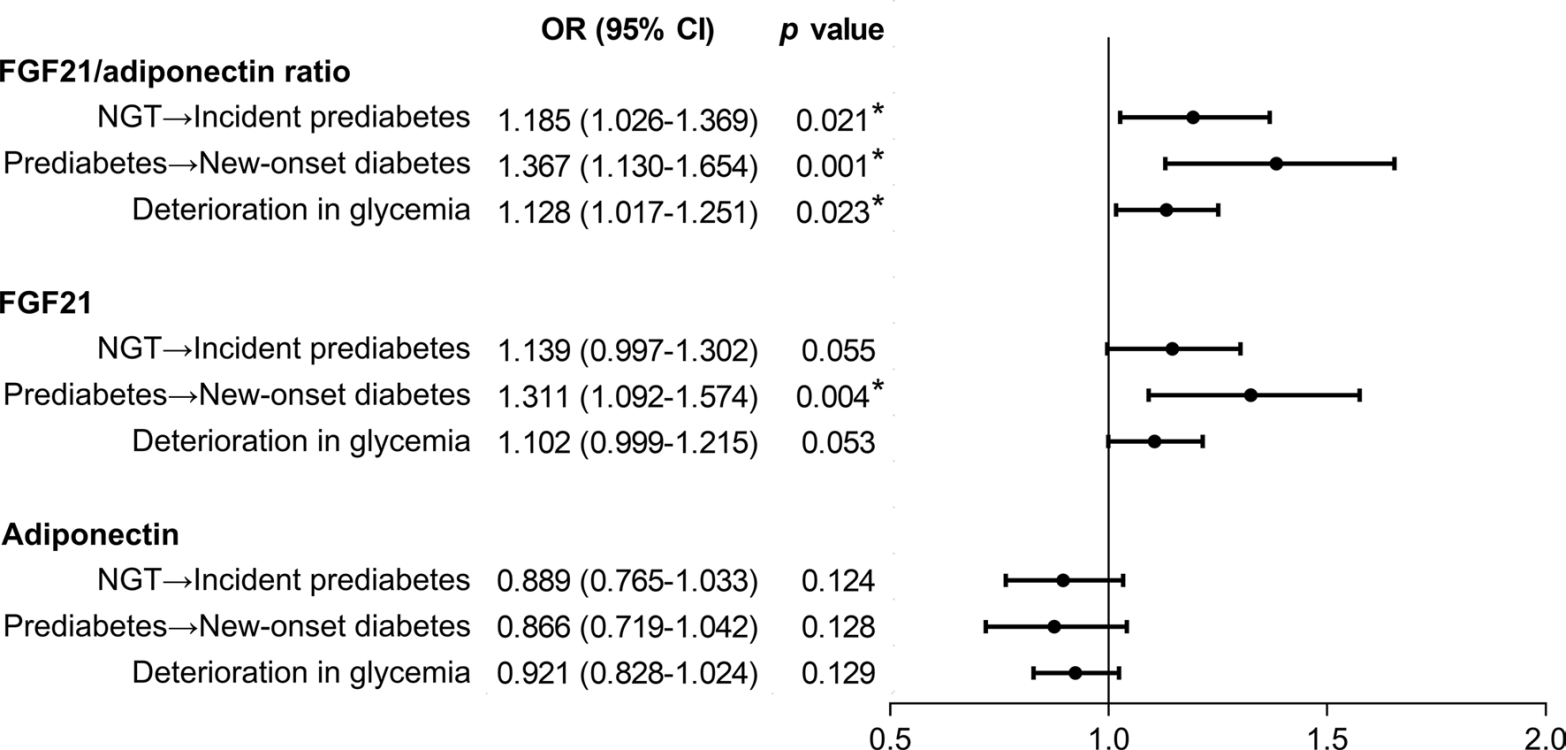
Associations of baseline FGF21/adiponectin ratio, FGF21, and adiponectin levels with incident prediabetes, new-onset diabetes, and deterioration in glycemia over a 4.6-year follow-up period. Odds ratios (95% confidence intervals) were obtained from multiple logistic regression analysis, referring to 1 SD change in log_e_-transformed serum FGF21/adiponectin ratio, FGF21, and adiponectin levels, and were adjusted for age, body mass index, waist circumference, systolic blood pressure, triglyceride, high- and low-density lipoprotein, high-sensitivity C-reactive protein, sex, homeostasis model assessment of insulin resistance, and HbA_1c_. *p value < 0.05. CI: confidence interval; FGF21: fibroblast growth factor 21; NGT: normal glucose tolerance; OR: odds ratio

Adiponectin is a powerful risk marker for incident prediabetes [22]. Therefore, the association between serum biomarkers and incident prediabetes was investigated in the current study. FGF21/adiponectin ratio was a predictor of incident prediabetes (OR 1.185 [95% CI 1.026–1.369], *p* = 0.021) independent of traditional risk factors, whereas FGF21 and adiponectin alone did not significantly predict incident prediabetes (both *p* > 0.05) (Fig. 1). Moreover, FGF21/adiponectin ratio independently predicted new-onset diabetes in subjects with prediabetes (OR 1.367 [95% CI 1.130–1.654], *p* = 0.001) (Fig. 1), no matter in males and females (Additional file 1: Table S5). There was a significant association of FGF21/adiponectin ratio with deterioration in glycemia (OR 1.128 [95% CI 1.017–1.251], *p* = 0.023), while neither FGF21 nor adiponectin levels were independent predictors of glycemic deterioration (Fig. 1).

Age is an important influencing factor associated with circulating adiponectin levels, and serum adiponectin levels are significantly higher in populations over 60–65 years old [23, 24]. We studied the association of FGF21/adiponectin ratio with new-onset diabetes in middle-age group and elderly group (< 60 years and ≥ 60 years). Baseline FGF21/adiponectin ratio levels were predictive of new-onset diabetes in both age groups (Additional file 1: Table S6).

### Baseline FGF21/adiponectin ratio was a superior biomarker to FGF21 or adiponectin alone

We further investigated whether FGF21/adiponectin ratio could be a clinically useful biomarker for diabetes prediction, using C statistics, NRI, and IDI. As shown in Table 4, serum FGF21/adiponectin ratio, FGF21, and adiponectin did not yield a significant improvement in C statistics (all *p* > 0.05) when added to Models 1, 2, or 3, including age, BMI, waist circumference, SBP, triglyceride, HDL-C, LDL-C, hsCRP, sex, HOMA-IR, and FPG (in Model 1), 2hPG (in Model 2), and HbA_1c_ (in Model 3). However, the addition of FGF21/adiponectin ratio to Model 1 resulted in a significant improvement in predicting new-onset diabetes, with increments in NRI (27.1%, *p* < 0.001) and IDI (0.7%, *p* = 0.020). There were similar increments in NRI and IDI when FGF21/adiponectin ratio levels were added to Models 2 and 3. Notably, we found that FGF21/adiponectin ratio provided an improvement in diabetes risk prediction, compared with the use of FGF21 (NRI = 23.5%, *p* = 0.002; IDI = 0.3%, *p* = 0.014) or adiponectin (NRI = 18.3%, *p* = 0.016; IDI = 0.3%, *p* = 0.161) alone.

**Table 4:** Comparison of incremental values between FGF21/adiponectin ratio and the use of FGF21 or adiponectin alone (n = 4797)

	AUC	95% CI	C statistics	NRI (%)	*p* value	IDI (%)	*p* value	C statistics	NRI (%)	*p* value	IDI (%)	*p* value
Model 1	0.791	0.756–0.826	Ref.	Ref.	Ref.	Ref.	Ref.					
+ FGF21/adiponectin ratio^a^	0.800	0.766–0.833	0.731	27.1	< 0.001	0.7	0.020	Ref.	Ref.	Ref.	Ref.	Ref.
+ FGF21^a^	0.798	0.765–0.832	0.772	17.4	0.021	0.4	0.101	0.955	− 23.5	0.002	− 0.3	0.014
+ Adiponectin^a^	0.793	0.758–0.828	0.939	23.9	0.001	0.3	0.015	0.789	− 18.3	0.016	− 0.3	0.161
Model 2	0.744	0.708–0.780	Ref.	Ref.	Ref.	Ref.	Ref.					
+ FGF21/adiponectin ratio^a^	0.756	0.720–0.791	0.643	32.7	< 0.001	0.6	0.003	Ref.	Ref.	Ref.	Ref.	Ref.
+ FGF21^a^	0.755	0.720–0.790	0.670	30.6	< 0.001	0.5	0.008	0.967	− 11.9	0.114	− 0.1	0.262
+ Adiponectin^a^	0.746	0.710–0.782	0.924	14.6	0.052	0.1	0.074	0.714	− 31.3	< 0.001	− 0.5	0.007
Model 3	0.818	0.782–0.853	Ref.	Ref.	Ref.	Ref.	Ref.					
+ FGF21/adiponectin ratio^a^	0.821	0.785–0.857	0.894	26.9	< 0.001	0.6	0.050	Ref.	Ref.	Ref.	Ref.	Ref.
+ FGF21^a^	0.821	0.785–0.856	0.897	19.8	0.009	0.4	0.083	0.997	− 14.7	0.051	− 0.1	0.212
+ Adiponectin^a^	0.818	0.782–0.853	0.999	18.9	0.012	0.2	0.088	0.896	− 19.8	0.008	− 0.4	0.100

Model 1 included baseline age, BMI, waist circumference, SBP, triglyceride, HDL-C, LDL-C, hsCRP, sex, HOMA-IR, FPG

Model 2 included baseline age, BMI, waist circumference, SBP, triglyceride, HDL-C, LDL-C, hsCRP, sex, HOMA-IR, 2hPG

Model 3 included baseline age, BMI, waist circumference, SBP, triglyceride, HDL-C, LDL-C, hsCRP, sex, HOMA-IR, HbA_1c_

Values above zero for the NRI and IDI indicate improved risk prediction and discrimination with the addition of biomarkers to the model

2hPG: 2-h plasma glucose; BMI: body mass index; FGF21: fibroblast growth factor 21; FPG: fasting plasma glucose; HDL-C: high-density lipoprotein cholesterol; HOMA-IR: homeostasis model assessment of insulin resistance; hsCRP: high-sensitivity C-reactive protein; IDI: integrated discrimination improvement; LDL-C: low-density lipoprotein cholesterol; NRI: net reclassification improvement; Ref.: referent; SBP: systolic blood pressure

^a^Log_e_-transformed before analysis

## Discussion

In this study, we provide the first observation that baseline FGF21/adiponectin ratio levels are increased in diabetes and could be a strong predictor of deterioration in glycemia over a median follow-up of 4.6 years, independent of the classical risk factors including FPG, 2hPG, and HbA_1c_ [25, 26]. As a biomarker, FGF21/adiponectin ratio significantly improved the performance of the traditional risk prediction model for diabetes, with a predictive value that was superior to that of FGF21 or adiponectin alone.

Prediabetes is characterized by the presence of beta cell dysfunction and insulin resistance, which occur before glucose changes are detectable [3]. In our study, baseline FGF21/adiponectin ratio increased progressively with the degree of dysglycemia from NGT to prediabetes and diabetes, and it was positively correlated with FINS and HOMA-IR. FGF21/adiponectin ratio independently predicted new-onset diabetes in patients with prediabetes. Notably, a high FGF21/adiponectin ratio level was also associated with an increased risk of incident prediabetes among subjects with NGT, while neither FGF21 nor adiponectin were independent predictors of incident prediabetes. These findings support the notion that dysfunction of the FGF21-adiponectin pathway in adipose tissues occurs at an early stage of glucose metabolic deterioration and participates in the pathophysiology of diabetes in humans. Previous studies have demonstrated that FGF21 and adiponectin are involved in the regulation of insulin sensitivity [27–29], and disturbance of the FGF21-adiponectin pathway abrogates FGF21-induced improvement of insulin resistance [14]. Some clinical studies have reported the association of adiponectin with incident diabetes in subjects with IR, but not in those who were insulin-sensitive [30]. Additionally, the inverse relationship between adiponectin and incident diabetes was attenuated when further adjusted for the insulin sensitivity index [10]. These observations suggest that FGF21/adiponectin ratio is a strong predictor of glycemic deterioration, which may be attributed partially to insulin resistance.

The mechanism underlying the dysfunction of the FGF21-adiponectin pathway in insulin resistance remains unclear. Circulating FGF21 levels are increased in obesity and diabetes accompanied by disturbed expression of its receptor levels [9, 31, 32], which points to a resistance state. FGF21 resistance has been demonstrated in obese animals, and the poor response to exogenous FGF21 is correlated with the decreased expression of FGF21 receptors or co-receptor β-Klotho in FGF21-target tissues, such as the liver and white adipose tissue [33]. Interestingly, TNF-α-induced inflammation impaired β-Klotho expression and FGF21 responsiveness in adipocytes, while rosiglitazone reversed the downregulation of β-Klotho expression in vitro and in vivo [34]. Obesity is characterized by chronic low-grade inflammation [35], which is considered as a link between obesity, IR, and type 2 diabetes [36, 37]. FGF21 resistance impairs its ability to stimulate adiponectin secretion in adipose tissue, and chronic inflammation may mediate the relationship between the dysfunctional FGF21-adiponectin pathway and insulin resistance [13, 14]. A recent study found that exercise ameliorated the FGF21–adiponectin axis impairment in diet-induced obese mice, accompanied by the upregulation of β-Klotho, FGFR1, and FGFR2, and the inhibition of adipose tissue inflammation [38]. Additionally, the cJun NH2-terminal kinase (JNK) signaling pathway has been reported to participate in the FGF21-adiponectin axis [39]. Previous studies have demonstrated that JNK signaling is an important inflammatory pathway involved in insulin resistance [40, 41]. The role of chronic inflammation in the dysfunctional FGF21-adiponectin pathway requires further investigation. It is worth noting that circulating adiponectin levels are regulated by many other factors, such as oxidative stress, endoplasmic reticulum stress, and inflammation [42–44], which play an important role in the pathophysiology of diabetes. Therefore, the reduction in adiponectin levels regulated by other mechanisms also contributes to the elevation of serum FGF21/adiponectin ratio.

To our knowledge, this is the first large-scale prospective cohort study focusing on the association between FGF21/adiponectin ratio and deterioration in glycemia. We reported that FGF21/adiponectin ratio was an independent predictor of glycemic deterioration, including incident prediabetes and diabetes. However, there are several limitations to our study. First, not enough cases from NGT to new-onset diabetes were observed due to the relatively short follow-up time. In addition, OGTT was not conducted during the follow-up survey, which may have led to the potential exclusion of cases of new-onset diabetes and prediabetes. Nonetheless, the predictive value of FGF21/adiponectin ratio for deterioration in glycemia remains significant. Second, serum total adiponectin but not high-molecular-weight (HMW) adiponectin levels were measured in this study, while previous studies reported that total and HMW adiponectin concentrations were similarly associated with incident diabetes [45, 46]. Third, since this study was conducted in subjects aged 55–70 years in China, the findings could not be generalized to younger subjects and other ethnic populations. However, this may be of considerable value in the elderly population with a high prevalence of prediabetes [47]. Further prospective studies in other populations are warranted to validate the predictive value of FGF21/adiponectin ratio in type 2 diabetes.

## Conclusion

In summary, this prospective study in China revealed that FGF21/adiponectin ratio levels increased progressively with the degree of dysglycemia. Baseline FGF21/adiponectin ratio significantly predicted glycemic deterioration, with the potential to be a useful biomarker superior to FGF21 or adiponectin alone.

## Supplementary Information

**Additional file 1: Figure S1.** Flow diagram of the study population. **Table S1.** Correlations between baseline serum FGF21/adiponectin ratio and baseline risk factors (n = 6361). **Table S2.** Baseline and follow-up glucose metabolism of subjects without diabetes at baseline (n = 4797). **Table S3.** Baseline characteristics of subjects with or without incident prediabetes at 4.6 years (n = 1539). **Table S4.** Logistic regression analysis of baseline FGF21/adiponectin ratio, FGF21, and adiponectin levels in new-onset diabetes. **Table S5.** Logistic regression analysis of baseline FGF21/adiponectin ratio, FGF21, and adiponectin levels in new-onset diabetes among males and females with prediabetes (n = 3244). **Table S6.** Logistic regression analysis of baseline FGF21/adiponectin ratio, FGF21, and adiponectin levels in new-onset diabetes stratified by age (n = 4797).

## Data Availability

The datasets used and/or analyzed during the current study are available from the corresponding author on reasonable request.

## Abbreviations

2hPG: 2-h Plasma glucose
BMI: Body mass index
CI: Confidence interval
DBP: Diastolic blood pressure
FGF: Fibroblast growth factor
FINS: Fasting insulin
FPG: Fasting plasma glucose
HbA_1c_: Glycated hemoglobin A_1c_
HDL-C: High-density lipoprotein cholesterol
HMW: High-molecular-weight
HOMA: Homeostasis model assessment
hsCRP: High-sensitivity C-reactive protein
IDI: Integrated discrimination improvement
IR: Insulin resistance
LDL-C: Low-density lipoprotein cholesterol
NGT: Normal glucose tolerance
NRI: Net reclassification improvement
OGTT: Oral glucose tolerance test
OR: Odds ratio
SBP: Systolic blood pressure
