# Supplementary material for: FGF21/adiponectin ratio predicts deterioration in glycemia: a 4.6-year prospective study in China
Source: Cardiovasc Diabetol. 2021 Jul 28;20:157. doi: 10.1186/s12933-021-01351-1 (PMC8320224; doi:10.1186/s12933-021-01351-1)
Supplement: Supplementary file 1 — Additional file 1: Figure S1. Flow diagram of the study population. Table S1. Correlations between baseline serum FGF21/adiponectin ratio and baseline risk factors (n = 6361). Table S2. Baseline and follow-up glucose metabolism of subjects without diabetes at baseline (n = 4797). Table S3. Baseline characteristics of subjects with or without incident prediabetes at 4.6 years (n = 1539). Table S4. Logistic regression analysis of baseline FGF21/adiponectin ratio, FGF21, and adiponectin levels in new-onset diabetes. Table S5. Logistic regression analysis of baseline FGF21/adiponectin ratio, FGF21, and adiponectin levels in new-onset diabetes among males and females with prediabetes (n = 3244). Table S6. Logistic regression analysis of baseline FGF21/adiponectin ratio, FGF21, and adiponectin levels in new-onset diabetes stratified by age (n = 4797). [file 12933_2021_1351_MOESM1_ESM.docx]

**Figure S1.** Flow diagram of the study population

Participants included in the statistical analysis

n = 6361

Did not develop PreDM/DM

n = 1188

Developed DM

n = 14

Baseline prediabetes

n = 3244

Did not develop DM

n = 3063

Developed DM

n = 181

Non-DM at baseline

n = 4797

Participants in the baseline survey, 04/2013-08/2014

n = 17212

Participants in the follow-up survey, 05/2018-09/2018

n = 7230

Inclusion criteria:

Aged 45-70 years

Lived in Nicheng County ≥ 5 years

Inclusion criteria:

Aged 55-70 years at baseline

Excluded 687 participants without baseline serum samples

DM at baseline

n = 1564

Baseline NGT

n = 1553

Excluded 182 participants using thiazide diuretics, statins, antipsychotics, glucocorticoids, and insulin

Developed PreDM

n = 351

Abbreviations: DM, diabetes mellitus; NGT, normal glucose tolerance, preDM, prediabetes.

**Table S1. Correlations between baseline serum FGF21/adiponectin ratio and baseline risk factors (n = 6361)**

|  | **Correlation with FGF21/adiponectin ratio*^a^*** | | | | |
| --- | --- | --- | --- | --- | --- |
|  | **Unadjusted** |  |  | **Adjusted for age & BMI** | |
|  | **r** | ***p*** |  | **r** | ***p*** |
| Age (years) | -0.01 | 0.337 |  | — | — |
| BMI (kg/m^2^) | 0.26 | <0.001 |  | — | — |
| Males (%) | -0.07 | <0.001 |  | -0.07 | <0.001 |
| Current smoker (%) | 0.05 | <0.001 |  | 0.06 | <0.001 |
| Current Drinker (%) | 0.03 | 0.021 |  | 0.04 | 0.003 |
| Waist circumference (cm) | 0.26 | <0.001 |  | 0.09 | <0.001 |
| Systolic blood pressure (mmHg) | 0.10 | <0.001 |  | 0.05 | <0.001 |
| Diastolic blood pressure (mmHg) | 0.11 | <0.001 |  | 0.05 | <0.001 |
| Total cholesterol (mmol/L) | 0.02 | 0.086 |  | 0.00 | 0.974 |
| Triglyceride (mmol/L)*^a^* | 0.41 | <0.001 |  | 0.35 | <0.001 |
| HDL-C (mmol/L)*^a^* | -0.37 | <0.001 |  | -0.31 | <0.001 |
| LDL-C (mmol/L) | 0.06 | <0.001 |  | 0.02 | 0.069 |
| hsCRP (μg/mL)*^a^* | 0.21 | <0.001 |  | 0.15 | <0.001 |
| Fasting plasma glucose (mmol/L) | 0.12 | <0.001 |  | 0.09 | <0.001 |
| 2-hour plasma glucose (mmol/L) | 0.18 | <0.001 |  | 0.14 | <0.001 |
| Fasting insulin (μU/mL)*^a^* | 0.27 | <0.001 |  | 0.16 | <0.001 |
| HOMA-β (%)*^a^* | 0.17 | <0.001 |  | 0.08 | <0.001 |
| HOMA-IR*^a^* | 0.28 | <0.001 |  | 0.17 | <0.001 |
| HbA_1c_ (mmol/mol) | 0.11 | <0.001 |  | 0.07 | <0.001 |
| HbA_1c_ (%) | 0.11 | <0.001 |  | 0.07 | <0.001 |

*^a^* Log_e_-transformed before analysis.

Abbreviations: BMI, body mass index; FGF21, fibroblast growth factor 21; HDL-C, high-density lipoprotein cholesterol; HOMA, homeostasis model assessment; hsCRP, high-sensitivity C-reactive protein; IR, insulin resistance; LDL-C, low-density lipoprotein cholesterol.

**Table S2. Baseline and follow-up glucose metabolism of subjects without diabetes at baseline (n = 4797)**P据。ian

|  | **Baseline** | **Follow-up** | ***p* value** |
| --- | --- | --- | --- |
| **Did not develop prediabetes and diabetes (n = 4251)** |  |  |  |
| Fasting plasma glucose (mmol/L) | 5.76 ± 0.49 | 5.13 ± 0.49 | <0.001 |
| Fasting insulin (μU/mL)*^a^* | 6.47 (4.62,9.10) | 5.79 (3.86,8.76) | <0.001 |
| HOMA-β (%)*^a^* | 58.39 (41.14,81.93) | 74.60 (48.63,111.30) | <0.001 |
| HOMA-IR*^a^* | 1.66 (1.16,2.36) | 1.31 (0.86,2.02) | <0.001 |
| HbA_1c_ (mmol/mol) | 37.39 ± 3.95 | 37.84 ± 3.57 | <0.001 |
| HbA_1c_ (%) | 5.57 ± 0.36 | 5.61 ± 0.33 | <0.001 |
|  |  |  |  |
| **Developed prediabetes (n = 351)** |  |  |  |
| Fasting plasma glucose (mmol/L) | 5.55 ± 0.44 | 5.12 ± 0.49 | <0.001 |
| Fasting insulin (μU/mL)*^a^* | 5.49 (4.07,7.67) | 5.35 (3.46,8.51) | 0.239 |
| HOMA-β (%)*^a^* | 53.12 (40.01,74.22) | 68.98 (45.11,106.01) | <0.001 |
| HOMA-IR*^a^* | 1.36 (1.00,1.91) | 1.22 (0.75,2.02) | <0.001 |
| HbA_1c_ (mmol/mol) | 36.01 ± 1.85 | 39.82 ± 1.57 | <0.001 |
| HbA_1c_ (%) | 5.44 ± 0.17 | 5.79 ± 0.14 | <0.001 |
|  |  |  |  |
| **Developed diabetes (n = 195)** |  |  |  |
| Fasting plasma glucose (mmol/L) | 6.21 ± 0.45 | 6.56 ± 1.36 | <0.001 |
| Fasting insulin (μU/mL)*^a^* | 7.91 (5.51,11.27) | 8.66 (4.98,14.65) | 0.006 |
| HOMA-β (%)*^a^* | 60.28 (39.60,84.53) | 60.19 (33.10,101.11) | 0.239 |
| HOMA-IR*^a^* | 2.16 (1.48,3.22) | 2.42 (1.43,4.28) | 0.001 |
| HbA_1c_ (mmol/mol) | 41.46 ± 3.96 | 48.67 ± 8.70 | <0.001 |
| HbA_1c_ (%) | 5.94 ± 0.36 | 6.61 ± 0.80 | <0.001 |

Data are presented as mean ± SD or median (interquartile range).

*^a^* Log_e_-transformed before analysis.

Abbreviations: HOMA, homeostasis model assessment; IR, insulin resistance.

**Table S3. Baseline characteristics of subjects with or without incident prediabetes at 4.6 years (n = 1539)**

|  | Without incident prediabetes | With incident prediabetes | *p* value |
| --- | --- | --- | --- |
| n | 1188 | 351 |  |
| Males (%) | 48.48 | 44.44 | 0.183 |
| Age (years) | 61.13 ± 3.84 | 61.83 ± 4.05 | 0.003 |
| Current smoker (%) | 25.17 | 24.22 | 0.717 |
| Current Drinker (%) | 16.50 | 15.38 | 0.619 |
| BMI (kg/m^2^) | 23.92 ± 2.97 | 24.15 ± 3.09 | 0.197 |
| Waist circumference (cm) | 82.61 ± 8.92 | 83.54 ± 9.41 | 0.092 |
| Systolic blood pressure (mmHg) | 132.04 ± 15.52 | 133.23 ± 15.93 | 0.210 |
| Diastolic blood pressure (mmHg) | 83.18 ± 8.64 | 83.58 ± 9.70 | 0.460 |
| Total cholesterol (mmol/L) | 5.04 ± 0.95 | 5.09 ± 0.94 | 0.341 |
| Triglyceride (mmol/L)*^a^* | 1.15 (0.81,1.64) | 1.14 (0.83,1.58) | 0.522 |
| HDL-C (mmol/L)*^a^* | 1.33 (1.09,1.59) | 1.36 (1.11,1.62) | 0.574 |
| LDL-C (mmol/L) | 2.96 ± 0.75 | 3.02 ± 0.78 | 0.200 |
| hsCRP (μg/mL)*^a^* | 0.67 (0.36,1.33) | 0.81 (0.38,1.53) | 0.076 |
| Fasting plasma glucose (mmol/L) | 5.46 ± 0.35 | 5.55 ± 0.44 | <0.001 |
| 2-hour plasma glucose (mmol/L) | 6.14 ± 1.10 | 6.35 ± 0.99 | 0.001 |
| Fasting insulin (μU/mL)*^a^* | 5.50 (3.99,7.67) | 5.49 (4.07,7.67) | 0.952 |
| HOMA-β (%)*^a^* | 56.44 (41.26,77.26) | 53.12 (40.01,74.22) | 0.058 |
| HOMA-IR*^a^* | 1.34 (0.96,1.89) | 1.36 (1.00,1.91) | 0.569 |
| HbA_1c_ (mmol/mol) | 34.16 ± 2.95 | 36.01 ± 1.85 | <0.001 |
| HbA_1c_ (%) | 5.28 ± 0.27 | 5.44 ± 0.17 | <0.001 |
| FGF21 (pg/mL)*^a^* | 200.33 (117.03,302.62) | 213.28 (128.91,329.95) | 0.148 |
| Adiponectin (μg/mL)*^a^* | 4.55 (3.35,6.08) | 4.50 (3.33,5.70) | 0.544 |
| FGF21/adiponectin ratio (pg/μg)*^a^* | 44.86 (23.55,76.36) | 47.28 (25.64,86.95) | 0.164 |

Data are presented as mean ± SD or median (interquartile range).

*^a^* Log_e_-transformed before analysis.

Abbreviations: BMI, body mass index; FGF21, fibroblast growth factor 21; HDL-C, high-density lipoprotein cholesterol; HOMA, homeostasis model assessment of insulin resistance; IR, insulin resistance; hsCRP, high-sensitivity C-reactive protein; LDL-C, low-density lipoprotein cholesterol.

**Table S4. Logistic regression analysis of baseline FGF21/adiponectin ratio, FGF21, and adiponectin levels in new-onset diabetes**

| Model | Baseline variable | OR (95%CI) |  |  |
| --- | --- | --- | --- | --- |
|  |  | **FGF21/adiponectin ratio** | **FGF21** | **Adiponectin** |
| Males (n = 2107) |  |  |  |  |
| Model 1 |  |  |  |  |
| A | + FGF21/adiponectin ratio*^a^* | 1.40 (1.08−1.81) |  |  |
| B | + FGF21*^a^* |  | 1.28 (1.01−1.63) |  |
| C | + Adiponectin*^a^* |  |  | 0.77 (0.59−1.02) |
| Model 2 |  |  |  |  |
| A | + FGF21/adiponectin ratio*^a^* | 1.35 (1.05−1.74) |  |  |
| B | + FGF21*^a^* |  | 1.27 (1.01−1.61) |  |
| C | + Adiponectin*^a^* |  |  | 0.82 (0.63−1.06) |
| Model 3 |  |  |  |  |
| A | + FGF21/adiponectin ratio*^a^* | 1.34 (1.04−1.72) |  |  |
| B | + FGF21*^a^* |  | 1.27 (1.00−1.61) |  |
| C | + Adiponectin*^a^* |  |  | 0.83 (0.65−1.08) |
|  |  |  |  |  |
| Females (n = 2690) |  |  |  |  |
| Model 1 |  |  |  |  |
| A | + FGF21/adiponectin ratio*^a^* | 1.46 (1.13−1.88) |  |  |
| B | + FGF21*^a^* |  | 1.41 (1.10−1.81) |  |
| C | + Adiponectin*^a^* |  |  | 0.84 (0.66−1.08) |
| Model 2 |  |  |  |  |
| A | + FGF21/adiponectin ratio*^a^* | 1.44 (1.12−1.86) |  |  |
| B | + FGF21*^a^* |  | 1.44 (1.13−1.84) |  |
| C | + Adiponectin*^a^* |  |  | 0.93 (0.73−1.18) |
| Model 3 |  |  |  |  |
| A | + FGF21/adiponectin ratio*^a^* | 1.31 (1.00−1.72) |  |  |
| B | + FGF21*^a^* |  | 1.30 (1.00−1.68) |  |
| C | + Adiponectin*^a^* |  |  | 0.94 (0.73−1.22) |
|  |  |  |  |  |
| Total (n = 4797) |  |  |  |  |
| Model 1 |  |  |  |  |
| A | + FGF21/adiponectin ratio>cutoff value | 1.93 (1.38−2.69) |  |  |
| B | + FGF21>cutoff value |  | 2.21 (1.59−3.09) |  |
| C | + Adiponectin>cutoff value |  |  | 0.63 (0.46−0.88) |
| Model 2 |  |  |  |  |
| A | + FGF21/adiponectin ratio>cutoff value | 1.90 (1.36−2.64) |  |  |
| B | + FGF21>cutoff value |  | 2.20 (1.58−3.05) |  |
| C | + Adiponectin>cutoff value |  |  | 0.71 (0.51−0.98) |
| Model 3 |  |  |  |  |
| A | + FGF21/adiponectin ratio>cutoff value | 1.77 (1.26−2.48) |  |  |
| B | + FGF21>cutoff value |  | 1.96 (1.39−2.75) |  |
| C | + Adiponectin>cutoff value |  |  | 0.70 (0.50−0.97) |

Model 1 included baseline age, BMI, waist circumference, SBP, triglyceride, HDL-C, LDL-C, hsCRP, sex (except for males and females), HOMA-IR, FPG

Model 2 included baseline age, BMI, waist circumference, SBP, triglyceride, HDL-C, LDL-C, hsCRP, sex (except for males and females), HOMA-IR, 2hPG

Model 3 included baseline age, BMI, waist circumference, SBP, triglyceride, HDL-C, LDL-C, hsCRP, sex (except for males and females), HOMA-IR, HbA_1c_

Data are presented as OR (95% CI). ORs are estimated as per SD increase.

*^a^* Log_e_-transformed before analysis.

Cutoff values of biomarkers are as followed: 73.96 pg/μg in males and 80.63 pg/μg in females for FGF21/adiponectin ratio; 393.59 pg/mL for FGF21; 3.06 μg/mL in males and 4.20 μg/mL in females for adiponectin.

Abbreviations: 2hPG, 2-hour plasma glucose; BMI, body mass index; FGF21, fibroblast growth factor 21; FPG, fasting plasma glucose; HDL-C, high-density lipoprotein cholesterol; HOMA-IR, homeostasis model assessment of insulin resistance; hsCRP, high-sensitivity C-reactive protein; LDL-C, low-density lipoprotein cholesterol; SBP, systolic blood pressure.

**Table S5. Logistic regression analysis of baseline FGF21/adiponectin ratio, FGF21, and adiponectin levels in new-onset diabetes among males and females with prediabetes (n = 3244)**

| Prediabetes patients | FGF21/adiponectin ratio | FGF21 | Adiponectin |
| --- | --- | --- | --- |
| Males | 1.38 (1.05-1.82) | 1.22 (0.94-1.57) | 0.70 (0.52-0.95) |
| Females | 1.47 (1.14-1.90) | 1.46 (1.14-1.88) | 0.88 (0.69-1.13) |

Data are presented as OR (95% CI). ORs are from multiple logistic regression analysis, referring to 1 SD change in log_e_-transformed serum FGF21/adiponectin ratio, FGF21, and adiponectin levels, and are adjusted for age, body mass index, waist circumference, systolic blood pressure, triglyceride, high- and low-density lipoprotein, high-sensitivity C-reactive protein, sex, homeostasis model assessment of insulin resistance, and fasting plasma glucose.

**Table S6. Logistic regression analysis of baseline FGF21/adiponectin ratio, FGF21, and adiponectin levels in new-onset diabetes stratified by age (n = 4797)**

| Age group | FGF21/adiponectin ratio | FGF21 | Adiponectin |
| --- | --- | --- | --- |
| < 60 years | 1.51 (1.12 - 2.03) | 1.41 (1.07 - 1.86) | 0.79 (0.58 - 1.07) |
| ≥ 60 years | 1.44 (1.14 - 1.80) | 1.34 (1.07 - 1.66) | 0.81 (0.65 - 1.02) |

Data are presented as OR (95% CI). ORs are from multiple logistic regression analysis, referring to 1 SD change in log_e_-transformed serum FGF21/adiponectin ratio, FGF21, and adiponectin levels, and are adjusted for age, body mass index, waist circumference, systolic blood pressure, triglyceride, high- and low-density lipoprotein, high-sensitivity C-reactive protein, sex, homeostasis model assessment of insulin resistance, and fasting plasma glucose.
